# Female Pattern Hair Loss in Female and Male: A Quantitative Trichoscopic Analysis in Chinese Han Patients

**DOI:** 10.3389/fmed.2021.649392

**Published:** 2021-03-26

**Authors:** Xi Chen, Xiangqian Li, Baifu Chen, Yue Yin, Jianzhong Zhang, Cheng Zhou

**Affiliations:** ^1^Department of Dermatology, Peking University People's Hospital, Beijing, China; ^2^Department of Dermatology, Beijing Tongren Hospital, Capital Medical University, Beijing, China

**Keywords:** hair, trichoscopy, female pattern hair loss, Chinese, gender

## Abstract

**Objectives:** To investigate the trichoscopic features of female pattern hair loss (FPHL) in Chinese Han patients and analyze the difference between male and female patients with FPHL.

**Materials and Methods:** Trichoscopic images were taken in four different scalp areas, including right frontal hairline, vertex, right parietal and occipital areas. Hair density, hair shaft diameter, vellus hair ratio and single hair follicle unit ratio were counted manually and analyzed.

**Results:** Seventy-three subjects were enrolled in this study, including 38 patients with FPHL (28 females and 10 males) and 35 normal controls without hair loss. The hair density and hair shaft diameter of FPHL patients reduced in the whole scalp. Vellus hair ratio and single hair follicle unit ratio were both increased in FPHL compared to normal controls. The vertex was the most affected area and the hair shaft diameter showed the most significant difference. Parietal and occipital area were also affected in FPHL. The reduction or increase was correlated with the severity of Ludwig staging. Very few gender differences were detected in male and female FPHL patients.

**Conclusion:** FPHL patients showed decreased hair density and hair shaft diameter, accompanied by increased vellus hair ratio and single hair follicle unit ratio. Parietal and occipital area can be also affected in FPHL, though not as severe as in vertex area. FPHL in male basically has the same characteristic as those in female patients.

**Limitation:** The main limitation of the study is the small sample size which only enrolled 10 male FPHL patients, in comparison to the female cases. The findings could not be representative of the normal population with the limited sample size.

## Introduction

Hair is one of the essential skin appendages in human. Thinning, damaging, or abnormal shedding of hair can not only impair the physical protection of scalp, but also affects mental health, and can even lead to severe social dysfunction. Androgenic alopecia (AGA) is the most common hair loss disease in the world, affecting both male and female. Rather than hair shedding, the process of hair loss in AGA is related to the gradual miniaturization of hair follicles, and eventually, lead to progressive, symmetric baldness (1). Two major hair loss patterns have been widely described in AGA. Male pattern hair loss (MPHL) or named male pattern baldness (MPB) usually manifests as a recession of the frontal hairline, with or without baldness of the vertex area (2). In contrast, female pattern hair loss (FPHL) presents with diffuse hair loss over the middle scalp and show no recession of frontal hairline, mainly affecting women (3).

The nomenclature of male and female pattern hair loss still cause confusion nowadays. Since the pathological changes of hair follicles are similar, most researchers regarded MPHL and FPHL as two variants of the same entity with different clinical presentation and treatment response (3, 4). It is hypothesized that FPHL and MPHL share common susceptibility genes, but genetic studies have not identified any susceptibility locus/gene for FPHL and suggested the etiology differs substantially from that of MPHL (5, 6).

Dermoscopy can magnify the skin lesions while eliminating the reflected light on the skin surface, helping clinicians to fully grasp the characteristics of disease skin lesions. As a branch of dermatoscopy, trichoscopy is cost-efficient, quick, and non-invasive, now is of great significance to evaluate hair and scalp disorders (7). When using a digital dermoscope (trichoscope), dermatologists can easily collect images with higher magnification, then use computer software to analyze the image data, such as hair density and hair shaft diameter. Previous studies have shown that one of the main trichoscopic features of AGA is the increased heterogeneity of hair shaft diameter, which can be quantified by hair shaft diameter and the ratio of vellus hair (8). The increase of single hair follicle units is also a manifestation of hair follicle microminiaturization in AGA patients (9). Besides, yellow dots, brown/white peripilar sign, honeycomb pigmentation, and pinpoint white dots are other important trichoscopic features of AGA, which are potentially related to the severity of the disease (10–12).

In quantitative trichoscpic evaluation, previous studies have analyzed several hair parameters of FPHL in female patients and have come to the consistent conclusion that miniaturization of hair follicles is the characteristics of FPHL in female, which is shown as reduced hair density and hair shaft diameter under trichoscopy (7, 13–15). Hair density and hair shaft diameter are the most used indicators in quantitation evaluation, and the percentage of vellus hair (<30 μm) has also been involved in the studies mentioned above. However, few researches have evaluated the percentage of single-hair follicle units, which is also of significance in FPHL diagnoses (7). In contrast to MPHL, in which occipital and parietal (side of the head) areas are usually not involved, parietal and occipital sites can also be affected in FPHL (14). At present, there still lacks a comprehensive evaluation of all four main hair parameters involving multiple scalp areas (frontal, vertex, parietal, and occipital areas) in FPHL.

Although FPHL is usually used to describe AGA in women, it can also be observed in a proportion of men (16, 17). With an overall AGA prevalence of 21.3% in Chinese male, 3.7% of the male patients showed FPHL type of hair loss (18). However, the trichoscopic feature of FPHL in male patients have not been well-studied yet (4). It is unclear whether the trichoscopic features of FPHL in male and female are the same or not.

In this study, we investigated the quantitative features of FPHL with digital trichoscope, and compared to the normal controls without hair loss. The differences between male and female FPHL patients were also analyzed.

## Materials and Methods

### Subjects

Both male and female Chinese Han subjects aged 18–59 years old who visited the dermatology department of Peking University People's hospital from January to August in 2020 were recruited into the study. The participants were screened by two experienced trichologists and categorized in two groups. The FPHL cases were diagnosed according to the Ludwig classification, with diffuse balding process and retention of the frontal hairline (3). The group of normal scalp subjects comprised volunteers who visited the dermatology clinic with complaints except for hair and scalp related diseases. The exclusion criteria included (1) presence of male pattern hair loss, alopecia areata, telogen effluvium, or other hair/scalp disorders; (2) positive results of hair-pull test; (3) history of anemia, thyroid disease, nutritional deficiency, autoimmune disease, or other systemic disease; (4) history of hair-loss treatment within 6 months; (5) hair coloring or perming within 3 months prior to the study; and (6) history of hair restoration surgery or scalp micropigmentation. All participants are agreed to participate in the study and provided written informed consent. The study was approved by the ethics committee of Peking University People's Hospital.

### Trichoscopy and Analysis

**Figure 1:**
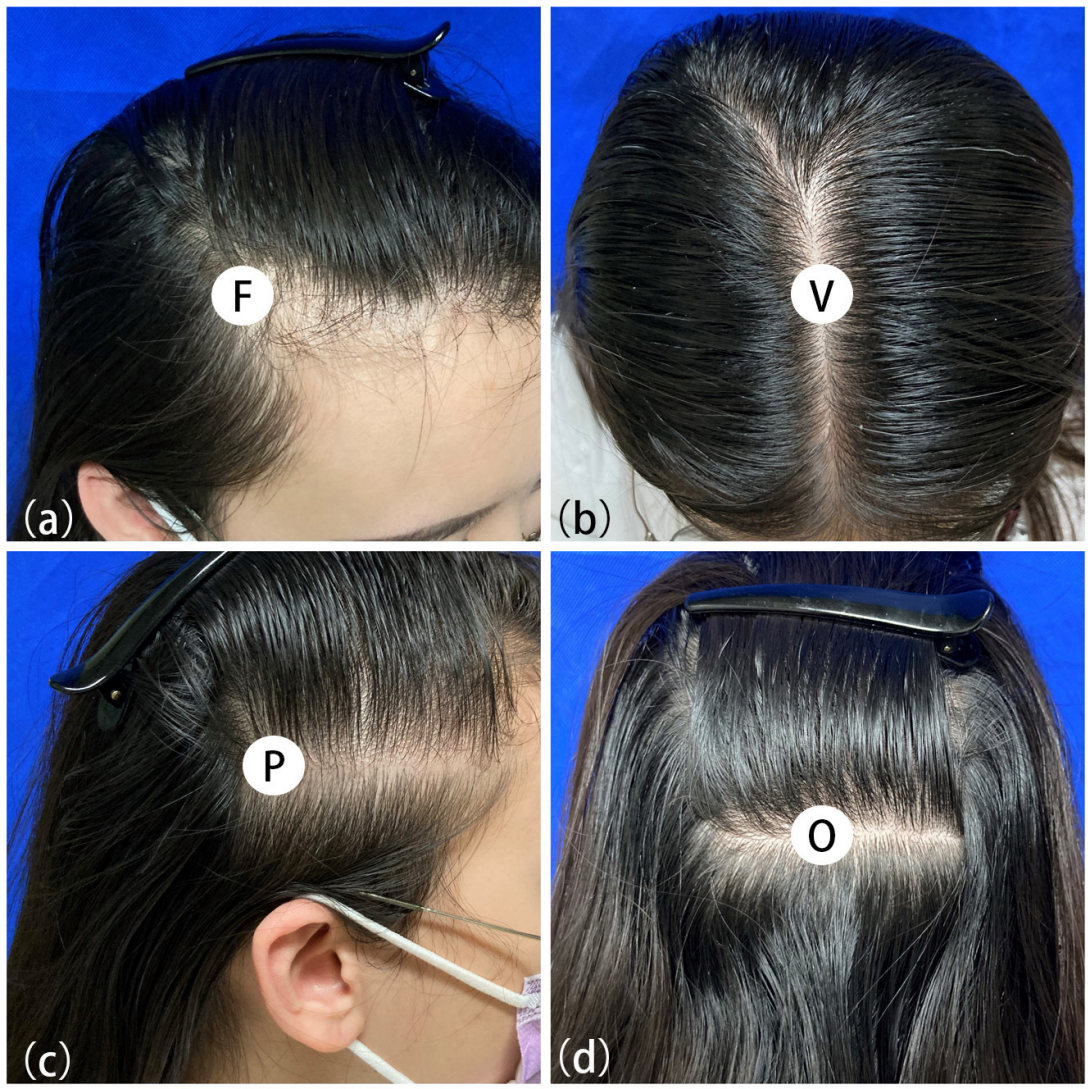
Measuring points of trichoscopy in frontal **(a)**, vertex **(b)**, parietal **(c)** and occipital **(d)** area.

Due to the symmetrical hair loss feature of FPHL, trichoscopy was performed in four regions, i.e., right frontal hairline, vertex, right parietal and occipital area, using a polarized contact dermoscope (Fotofinder body studio ATBM, FotoFinder Systems GmbH, Bad Birnbach, Germany). The measurement point of each area is shown in Figures 1a–d: (1) the top of right frontotemporal angle (marked as “frontal”); (2) the mid-point of the connection of the left and right external auditory ear canals (“vertex”); (3) 6 cm from the external ear canal on the right side of the participants (“parietal”); (4) the occipital carina (“occipital”). The lateral scalp is divided into three parts by hair transplant surgeons: temporal (anterior one-third), parietal (middle one-third), and occipital (posterior one-third). The measurement area of the lateral scalp used in this study may be called “temporal” or “temporoparietal” in some other previous researches; however, we named this point as “parietal” due to its anatomical location above the parietal bone.

In each area, one 20-fold and four 50-fold figures were taken. All the parameters were all manually measured in Trichoscale 3.0 software Smart Count (Fotofinder Universe 2019, Bad Birnbach, Germany). Hairs and hair follicle units in each 50-fold trichoscopic figure were noted and counted by clicking the mouse, which was conducted by two experienced trichologists. Hair shaft diameter was measured by mouse pointing and dragging vertically from one edge of the hair shaft to the other edge, and the average value was calculated.

### Statistical Analysis

Statistical analysis was done using SPSS software (IBM, 25.0, CA, USA). Narrative statistics described the basic characteristics of the population. Independent-sample *t*-tests and Chi-square tests were performed to test the significance between the FPHL patients and the normal subjects. Kruskal-Wallis *H*-tests, Mann-Whitney *U*-tests and Fisher's exact tests were performed to compare results between groups whose data did not subject to normal distribution. The results of the analysis were presented as mean ± standard deviation (SD), and a *p* < 0.05 was considered significant.

PASS 2019 software (NCSS LLC., Kaysville, U.T., USA) was used to calculate the sample size. The primary outcomes of this study are vertex hair density, hair-shaft diameter, vellus hair ratio and single hair follicle unit ratio of normal and FPHL patients. To detect the lowest mean difference of 15 with standard deviations of 19.0 and 11.0 between the two groups with a two-sided 0.0125% significance level (adjusted for multiple comparisons using Bonferroni method) and a power of 90%, a sample size of 33 for each group is needed, at last, 35 normal people and 38 FPHL people were enrolled in this study.

## Results

**Figure 2:**
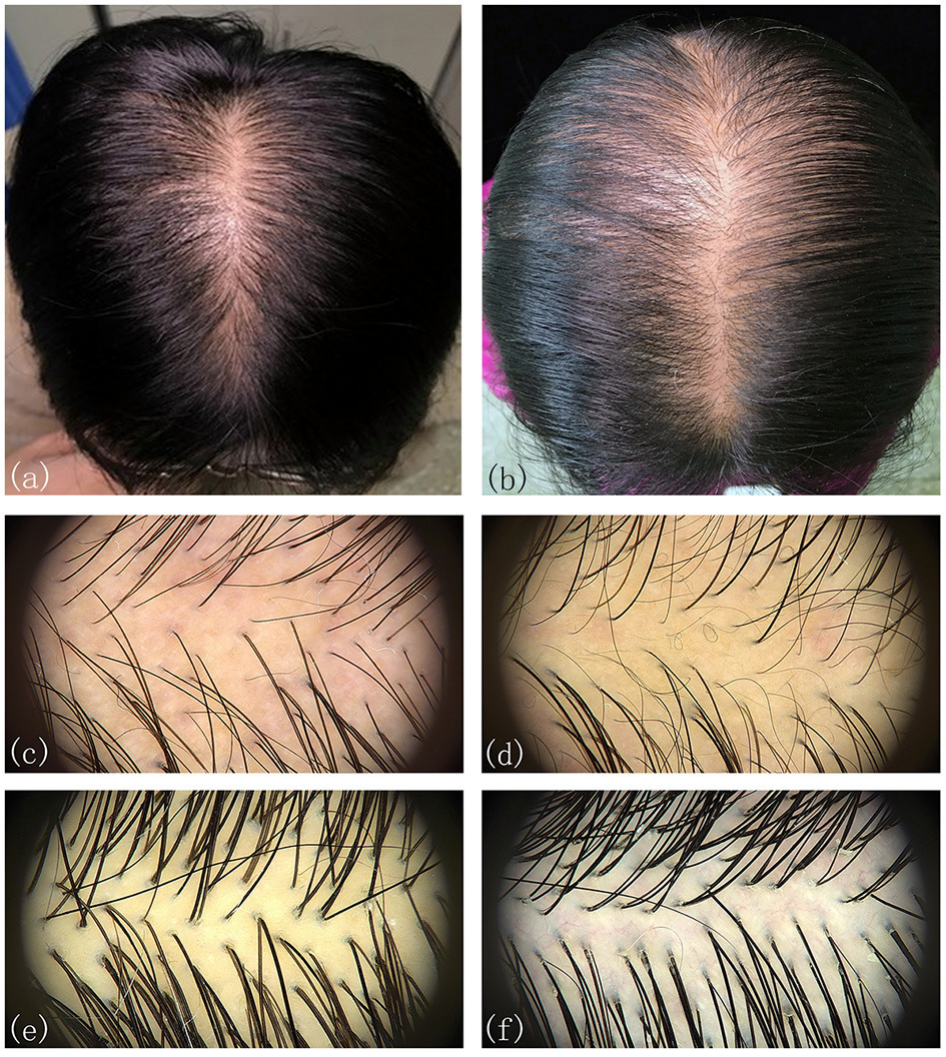
Typical clinical and trichoscopic feature (vertex) of male FPHL **(a,c)** and female FPHL **(b,d)** patients, compared with normal male **(e)**, and female **(f)**.

The participants were subdivided into two groups of 35 normal subjects and 38 FPHL patients based on the clinical diagnosis. The FPHL patients are graded following the Ludwig classification, from L1 to L3. The particular distribution of participants is shown in Table 1. The mean age of normal subjects and FPHL patients were 31.40 ± 10.38 and 31.08 ± 8.82 years old, respectively. No significant difference has been detected between normal and FPHL subjects. The typical clinical images of FPHL patients and trichoscopic images in vertex area of both FPHL and normal subjects are shown in Figure 2. The values of mean hair density, hair shaft diameter, vellus hair ratio and single hair follicle unit ratio at different scalp sites of FPHL patients are summarized in Tables 2, 3, and the parameters of normal male and female are shown in Table 4.

**Table 1:** Distribution of participants by hair loss status.

**Classification**		**Male**		**Female**		**Total**	
		***n***	**Age (year)**	***n***	**Age (year)**	***n***	**Age (year)**
FPHL	Ludwig L1	4	24.75 (± 3.43)	15	30.13 (± 7.96)	19	29.00 (± 7.51)
	Ludwig L2	4	29.75 (± 7.14)	9	35.78 (± 12.81)	13	33.92 (± 11.43)
	Ludwig L3	2	34.00 (± 1.41)	4	30.25 (± 5.44)	6	31.50 (± 4.68)
	Total	10	28.60 (± 5.89)	28	31.96 (± 9.59)	38	31.08 (± 8.21)
Normal		17	29.76 (± 11.02)	18	32.94 (± 9.79)	35	30.40 (± 10.38)
*p*-value		/	0.786	/	0.752	/	0.769

**Table 2:** Hair parameters of FPHL patients and normal subjects.

**Scalp area**	**Hair density (/cm**^****2****^**)**			**Hair shaft diameter (μm)**			**Vellus hair ratio (%)**			**Single hair follicle unit ratio (%)**		
	**FPHL**	**Normal**	***p*-value**	**FPHL**	**Normal**	***p*-value**	**FPHL**	**Normal**	***p*-value**	**FPHL**	**Normal**	***p*-value**
Frontal	102.89 (± 20.19)	117.07 (± 21.49)	0.010^*^	55.71 (± 8.83)	61.73 (± 6.92)	0.001^*^	22.00 (± 7.88)	16.89 (± 6.43)	0.003^*^	47.43 (± 16.82	35.78 (± 15.53)	0.005^*^
Vertex	112.05 (± 25.56)	140.21 (± 23.91)	<0.001^*^	54.23 (± 9.08)	67.38 (± 7.59)	<0.001^*^	20.92 (± 9.74)	9.26 (± 4.59)	<0.001^*^	39.69 (± 18.38	18.86 (± 11.37)	<0.001^*^
Parietal	85.18 (± 27.48)	96.19 (± 21.63)	0.028^*^	59.91 (± 10.02)	67.62 (± 8.30)	0.001^*^	16.68 (± 10.71)	10.72 (± 5.68)	0.001^*^	34.66 (± 18.26	17.89 (± 11.60)	<0.004^*^
Occipital	106.66 (± 22.94)	127.18 (± 22.7)	<0.001^*^	60.78 (± 10.31)	68.34 (± 7.70)	<0.001^*^	15.04 (± 8.2)	8.88 (± 4.23)	0.004^*^	28.32 (± 16.6)	13.47 (± 8.74)	<0.001^*^

* *p < 0.05*.

**Table 3:** Comparisons of hair parameters in FPHL patients between male and female.

**Scalp area**	**Hair density (/cm**^****2****^**)**			**Hair shaft diameter (μm)**			**Vellus hair ratio (%)**			**Single hair follicle unit ratio (%)**		
	**Male**	**Female**	***p*-value**	**Male**	**Female**	***p*-value**	**Male**	**Female**	***p*-value**	**Male**	**Female**	***p*-value**
Frontal	92.79 (± 21.29)	106.5 (± 18.87)	0.060	52.05 (± 8.29)	57.02 (± 8.79)	0.088	22.49 (± 5.74)	21.82 (± 8.6)	0.782	49.89 (± 24.29)	46.54 (± 13.71)	0.935
Vertex	113.1 (± 28.84)	111.68 (± 24.85)	0.883	53.23 (± 9.49)	54.59 (± 9.08)	0.636	18.15 (± 7.76)	21.91 (± 10.29)	0.302	38.3 (± 20.87)	40.18 (± 17.81)	0.807
Parietal	77.28 (± 10.14)	88.00 (± 31.14)	0.142	64.72 (± 9.63)	58.19 (± 9.74)	0.194	11.89 (± 8.35)	18.39 (± 11.07)	0.044^*^	34.32 (± 17.36)	34.78 (± 18.88)	0.568
Occipital	95.78 (± 16.38)	110.55 (± 23.92)	0.101	61.08 (± 11.32)	60.68 (± 10.14)	0.732	14.32 (± 8.39)	15.29 (± 8.27)	0.568	33.45 (± 20.65)	26.48 (± 14.9)	0.368

* *p < 0.05*.

**Table 4:** Hair parameter of normal male and female subjects.

**Scalp area**	**Hair density (/cm**^****2****^**)**			**Hair shaft diameter (μm)**			**Vellus hair ratio (%)**			**Single hair follicle unit ratio (%)**		
	**Male**	**Female**	***p*-value**	**Male**	**Female**	***p*-value**	**Male**	**Female**	***p*-value**	**Male**	**Female**	***p*-value**
Frontal	114.34 (± 24.12)	119.63 (± 19.01)	0.546	62.68 (± 5.04)	60.82 (± 8.37)	0.318	16.75 (± 5.98)	17.01 (± 6.99)	0.910	39.32 (± 16.40)	14.31 (± 8.37)	0.258
Vertex	137.63 (± 25.44)	142.63 (± 22.82)	0.503	68.03 (± 8.37)	66.74 (± 7.30)	0.590	9.00 (± 3.38)	9.49 (± 5.58)	0.752	18.86 (± 10.90)	18.87 (± 12.10)	0.909
Parietal	85.82 (± 17.23)^*^	105.98 (± 21.14)	0.004^*^	69.81 (± 7.51)	65.54 (± 8.67)	0.195	11.79 (± 5.18)	9.71 (± 6.09)	0.286	21.02 (± 13.64)	14.94 (± 8.68)	0.130
Occipital	127.51 (± 27.53)	126.87 (± 17.79)	0.883	68.96 (± 8.61)	67.75 (± 6.91)	0.443	10.07 (± 4.83)	7.72 (± 3.30)	0.105	15.83 (± 9.78)	7.20 (± 6.91)	0.163

* *p < 0.05*.

### Hair Density

The mean hair density at different scalp areas of our participants is shown in the first part of Tables 2–4. The hair density in parietal area was the lowest in both FPHL and normal participants, and in vertex area, all groups show the highest hair density (Tables 2, 3). When compared to the normal subjects, the hair density in FPHL patients tended to reduce and statistically significant differences were detected in all scalp areas, especially in vertex area (*p* < 0.0001). However, no gender difference of hair density was detected in FPHL patients (Table 3), though the hair density in parietal area of male normal subjects was significantly lower than female (Table 4).

### Hair Shaft Diameter

The second part of Tables 2–4 has shown the mean hair shaft diameter of male and female participants. In normal participants, the hair of the frontal area was the thinnest, while in the FPHL patients the vertex area showed the lowest hair shaft diameter (Table 2). The hair shaft diameter of FPHL patients decreased in the whole scalp with statistically significant differences compared to normal. The highest reduction of hair shaft diameter of FPHL patients was detected in the vertex area (*p* < 0.0001). No gender difference was detected whether in patients or normal (Tables 3, 4).

### Vellus Hair Ratio

The vellus hair ratio is shown in the third part of Tables 2–4. The frontal area had the highest vellus hair ratio and the lowest ratio was found in occipital area in both FPHL and normal participants. The vellus hair ratio showed a tendency of increase in FPHL patients when compared to normal, and significant differences were detected in all scalp areas (Table 2). In vertex area, the vellus hair ratio also showed the highest increase (*p* < 0.0001). In FPHL patients, minor gender difference in vellus hair ratio were detected in parietal area, though no difference was shown between normal male and female participants (Tables 3, 4).

### Single Hair Follicle Unit Ratio

The single hair follicle ratio is shown in the last part of Tables 2–4. Similar to the vellus hair ratio, the frontal area had the highest single hair follicle ratio and the lowest ratio was found in occipital area in both FPHL and normal participants (Table 2). All four areas in FPHL patients showed statistical significance compared to the normal subjects, and the vertex area still had the top decrease among other scalp areas (*p* < 0.0001). There was no gender difference in both normal and FPHL participants (Tables 3, 4).

### Hair Parameters From Grade L1 to L3 in FPHL Patients

**Figure 3:**
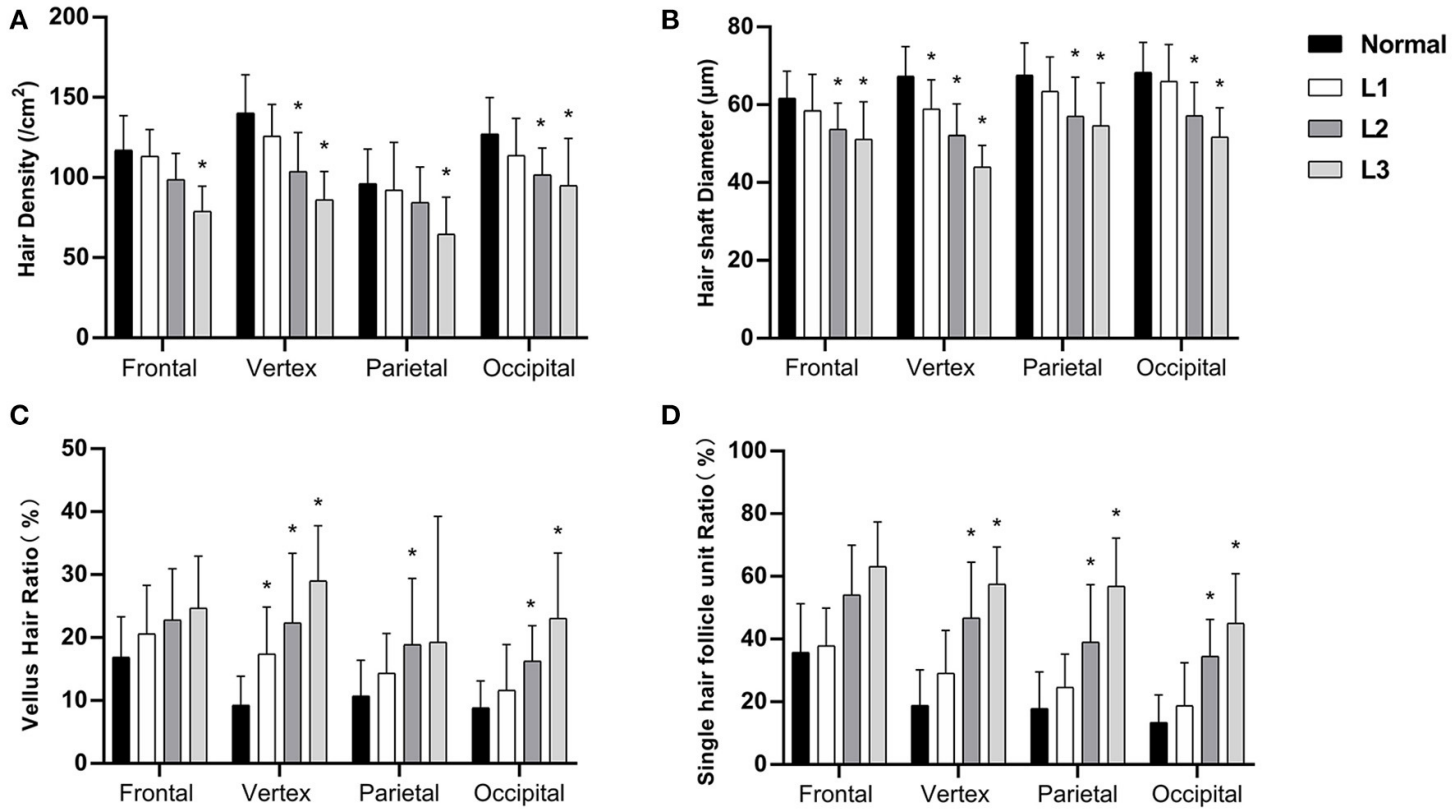
Hair density **(A)**, hair shaft diameter **(B)**, vellus hair ratio **(C)**, and single hair follicle unit ratio **(D)** of FPHL patients from Ludwig L1 to L3. *p < 0.05 compared with normal subjects.

We then analyzed the hair parameters of different severity in FPHL patients. According to the FPHL disease severity, patients were stratified into three groups (Ludwig L1-L3). In all scalp areas, the hair density and hair shaft diameter showed a decreasing tendency with the aggravation of the disease, and the ratio of vellus hair in and single hair follicle unit all raised as the disease got worse. Compared with normal group, the significant difference of the parameters was mainly found in vertex area (Figure 3) and in L2 and L3 groups. In L1 group, only the hair shaft diameter and vellus hair ratio of vertex area were significantly different with normal subjects.

## Discussion

According to our quantitative trichoscopic analysis, FPHL involved the whole scalp, especially at the vertex area, and primarily manifested as a decrease in hair shaft diameter. All of the four hair parameters including hair density, hair shaft diameter and the ratio of vellus hair and single hair follicle unit, have changed in our FPHL patients when compared to normal, confirmed that the miniaturization of hair follicle is the characteristics of FPHL. Similar to previous research, our study also showed that the involvement of parietal and occipital area is an additional important feature of FPHL (7, 13–15). This pattern of hair involvement is quite different from MPHL. The parietal and occipital parts are usually not involved in MPHL patients, and therefore occipital and even parietal parts are appropriate donor sites for hair transplantation (19). Thus, when consulting hair transplantation with FPHL patients, it should be fully considered that the hair may be abnormal in the whole scalp, and a comprehensive examination should be carried out. Medical treatment of parietal or occipital area is also necessary if the region has been involved. Besides, the frontal area was also involved slightly even though no receding of hairline was observed clinically.

The changes of the hair parameters were correlated with the severity of the disease, from Ludwig grade 1 to 3 (Figure 3), and the vertex area was the most severely affected area. In the early stage of disease (L1), significant decreases in hair shaft diameter had been detected and the vellus hair ratio also increased significantly. Although not as severe as the vertex area, all the parameters of parietal and occipital area also showed significant difference in the advanced stages of the disease (L2 and L3).

Though FPHL in male has been described previously (20), studies on the clinical features are extremely limited, especially on trichoscopic features. During a retrospective study of 2,140 male AGA patients, only 84 (3.9%) patients were diagnosed as FPHL (17).

FPHL in male has similar clinical manifestations as FPHL in female, showing diffuse scalp hair loss with no obvious recession of the frontal hairline (21). Compared with female patients in the present study, we found the hair parameters changed in male FPHL patients are similar to that in female FPHL patients. Importantly, in male FPHL patients, parietal and occipital area can also be involved, which pattern is totally different from MPHL. Therefore, in our experience, diagnosing these male FPHL patients as MPHL or MPB is not appropriate. We suggested that FPHL in male or female should be diagnosed based on different hair loss patterns and trichoscopic alterations, rather than on the basis of gender alone. It is important to establish a classification for assessment and grading of parietal and occipital involvement, which could overcome the limitations in the Ludwig classification, the Sinclair scale and the basic and specific (BASP) classification (3, 22, 23). Oral finasteride 1 mg/day is effective in the majority of male patients with AGA, while in female FPHL patients it could not significantly slow hair thinning, increase hair growth or improve the appearance of the hair unless increasing the dose to 2.5–5 mg/day (24, 25). Whether available treatments for male FPHL patients could be as efficacy as in MPHL is unclear, and further treatment studies on male FPHL patients are needed. This study provides some evidence for clarifying the term of FPHL in male and is of great value for further study on its pathogenesis, treatment, and the similarities and differences with MPHL.

The two parameters, hair density and hair shaft diameter, are trichoscopic features that need precisely quantitative measurement, which might not be practical for daily diagnostics purposes. In our experience, vellus hair ratio and single hair follicle unit ratio are more likely to be perceived and estimated under trichoscopy without quantitative measurements, which are more valuable in clinical practice.

During the progressive and unsynchronized miniaturization of hair follicles in AGA, terminal hairs are gradually replaced by intermediate hair and vellus hairs. Although the relevant quantitative research is relatively limited, researchers have long agreed that hair shaft thickness heterogeneity of more than 20% is a hallmark of AGA, which means that vellus hair percentage for more than 20% of all the hairs in the same view (10, 12). In our study, the vellus hair ratio of FPHL in frontal and vertex areas were both over 20%. In the parietal and occipital area of the FPHL scalp, vellus hair ratio also increased, but not as much as 20%. The present values appear to be similar to the previous study of Caucasian women (9). Mai et al. has also used “terminal/vellus hair ratio” to describe the vellus hair percentage in their study, and we found our results were basically consistent with each other after conversion (15). According to the hair involvement characteristics of FPHL, evaluation of vellus hair ratio should be established on different parts of the scalp.

Hair exists in the form of follicular units on the scalp. The hair follicle unit, also known as the pilosebaceous gland unit, is composed of 1–5 hairs, erect hair muscles, sebaceous glands, and peripheral nerves and blood vessels. Guo et al. indicated that the hair follicle unit types found in healthy Han Chinese are dominated by 2-hair follicle units, followed by single hair follicle units and 3-hair follicle units (26). The increase of single hair follicle units is also a trichoscopic manifestation of the miniaturization of hair follicles in AGA patients. Rakowska et al. included the increase of single hair follicle units as one of the secondary indicators of the trichoscopy criteria for diagnosing female androgenic alopecia (9). In our study, single hair follicle unit ratio elevated in FPHL patients as expected, however, it was not as high as in Caucasians. Interestingly, the single hair follicle unit ratio in Chinese normal controls was higher than Caucasians (9).

Hair density in this study showed a similar value to the previous study in Southern Chinese women (15). However, when comparing with other studies on Asian women including Korean and Thai women, the hair density of Chinese normal participants was a little bit lower and much lower than Caucasians, Hispanics and Africans (13, 27–29). In terms of hair shaft diameter, our results also seemed to be much “lower” than other similar studies except for the study of Caucasians women conducted by Rakowska et al. (9). The variation of the results in different studies may be caused by different devices and manners for the studies. Most of the previous study included about five randomly selected terminal hairs for hair shaft diameter measurement under 100-fold magnification, using trichoscopy system Folliscope® (LeedM Corporation) (13, 28). While in our study and Rakowska et al. (9) study, vellus hair was also included in the diameter measurement.

Gender factors showed a limited influence on hair parameters. Birnbaum et al. found that the differences in hair density of frontal, vertex and occipital area between men and women were not statistically significant in health Americans (including Hispanic, Caucasian, and African American) (27). A similar study of healthy Thai people also showed the same results (29). The only significant difference we noticed in this study is that the hair density of parietal region of men is lower than that of women, which was blank in the study of American people and not consistent with the data of Thai People. This gender difference in parietal hair density remains to be confirmed by more extensive studies.

## Limitation

Compared with other trichoscopic studies on FPHL, the sample size of this study was relatively small. The main limitation of the study cohort is the presence of only 10 male subjects with FPHL, in comparison to the female cases. Though the study could be able to detect the difference of trichoscopic features between FPHL and normal controls, the findings could not be representative of the normal population.

## Conclusion

In this study, the hair involvement in FPHL of Chinese Han patients was summarized with quantitative trichoscopic analysis. The miniaturization of hair follicles is the main characteristic of FPHL, which is shown as decreased hair density and hair shaft diameter, accompanied by increased vellus hair ratio and single hair follicle unit ratio. The hair in parietal and occipital area can be also affected, though not as severe as in vertex area. Although the incidence is exceptionally low, FPHL can occur in man, and its characteristics are basically the same as those in female patients.

## Data Availability Statement

The raw data supporting the conclusions of this article will be made available by the authors, without undue reservation.

## Ethics Statement

The studies involving human participants were reviewed and approved by Ethics Committee of Peking University People's Hospital. The patients/participants provided their written informed consent to participate in this study.

## Author Contributions

All authors listed have made a substantial, direct and intellectual contribution to the work, and approved it for publication.

## Conflict of Interest

The authors declare that the research was conducted in the absence of any commercial or financial relationships that could be construed as a potential conflict of interest.
